# The Oncopromoting Gene RBM6 Inhibits Prostate Tumour Cell Migration During Epithelial‐to‐Mesenchymal Transition

**DOI:** 10.1111/jcmm.70397

**Published:** 2025-02-03

**Authors:** Ruoyang Liu, Yu Liu, Long Zhang, Xiang Li, Ningyang Li, Fubo Lu, Wansheng Gao, Zhankui Jia, Zhenlin Huang, Jinjian Yang

**Affiliations:** ^1^ Department of Urology The First Affiliated Hospital of Zhengzhou University Zhengzhou Henan China; ^2^ Key Laboratory of Urinary Tumors Henan Provincial Health Commission Zhengzhou Henan China

**Keywords:** CDH1, prostate tumour, RBM6, tumour metastasis, ZEB1

## Abstract

RBM6, implicated in the progression of multiple tumour types but unexplored in prostate tumours, was found to indicate potential therapeutic implications due to its elevated expression in prostate tumours. To elucidate its molecular function, scratch tests, transwell migration and invasion assays were conducted, with PCR and western blot analyses verifying molecular regulatory relationships. RNA pulldown and RNA immunoprecipitation tests were also employed to investigate underlying mechanisms. Results indicate that RBM6 enhances prostate cell migration by suppressing CDH1, yet ZEB1 overexpression alleviates this suppression. Notably, under these conditions, RBM6's inhibitory effect on MMP16 becomes more pronounced, reducing cell migration ability. Thus, under normal conditions, RBM6 promotes prostate tumour cell migration, but in the context of high ZEB1 expression, it inhibits migration. This shift in RBM6's regulatory capacity towards downstream genes underscores the importance of considering objective conditions in studying RBM6 molecules.

## 1 Introduction

Prostate cancer is the second most common cancer in the world and the fifth leading cause of cancer death in men in 2022 [1]. Androgen deprivation therapy (ADT), as the standard treatment for advanced prostate cancer, can achieve good results [2]. While targeting AR in prostate cancer is already a translational success story, there will inevitably be resistance [3], these patients often have metastatic prostate cancer and are at high risk [4]. Therefore, exploring new potential therapeutic targets is particularly important for the treatment of prostate cancer, especially castration‐resistant prostate cancer.

During tumour progression, the tumour microenvironment often leads to the emergence of epithelial–mesenchymal transformation (EMT), which induces cell metastasis [5], Similarly, it has been reported that EMT can lead to partial treatment resistance when it occurs [6]. Advanced prostate castration‐resistant prostate cancer (CRPC) patients have always been the focus and difficulty in the treatment of prostate cancer. It has been reported that the synergistic use of taxane and AR inhibitors may overcome taxane resistance through the EMT–MET cycle mechanism [7, 8]. The study of the precursor pathway of EMT, combined with strategies to inhibit androgen signalling, is expected to increase the effectiveness of treatment regimens by inducing biochemical changes in reproduction.

Matrix metalloproteinase (MMP) family possess the capability to degrade nearly all protein constituents within the extracellular matrix (ECM), thereby dismantling the histological barriers that impede tumour cell invasion. Consequently, they play a pivotal role in facilitating tumour invasion and metastasis. As such, their significance in these oncogenic processes has garnered increasing attention, and they are recognised as the primary proteolytic enzymes driving these events [9]. It has been reported that MMP16 exhibits a close association with migration in certain types of tumours; however, its specific role in prostate tumours remains largely unexplored [10].

RNA‐binding protein (RBP) plays a key role in cell physiology by regulating the expression of target RNA transcripts after transcription [11]. The RNA‐binding motif (RBM) proteins are a class of RBPs named, containing RNA‐recognition motifs (RRMs), RNA‐binding domains, and ribonucleoprotein motifs [12]. RNA‐binding motif protein 6 (RBM6), as a member of the RBM family, has been reported to be associated with a variety of tumours, for example, RBM6 inhibits laryngocarcinoma but promotes lung cancer [13, 14], however its relationship with prostate tumours has been unclear.

This study found that RBM6 can promote the migration ability of prostate tumours through CDH1, and its expression is regulated by ZEB1 transcription. Interestingly, RBM6 inhibited prostate tumour migration when ZEB1 was highly expressed, and increased RBM6 inhibited tumour migration in enzalutamide‐resistant LNCaP cell lines.

## 2 Materials and Methods

### 2.1 Cell Lines, Cell Culture and Transfection

The DU145, PC‐3, LNCaP and 293T cell lines were obtained from the Cell Bank of the Chinese Academy of Sciences (Shanghai, China). PC‐3 and LNCaP cells were maintained in RPMI‐1640 medium, 293T cells were cultured in DMEM, and DU145 cells were grown in MEM, with all media supplemented with 10% FBS. The cells were cultured in the logarithmic growth phase. Transfections were carried out using Lipofectamine 2000 (Thermo Fisher Scientific), according to the manufacturer's instructions. The information the shRNA sequences is provided in Table S1.

### 2.2 Reverse Transcription–Quantitative Polymerase Chain Reaction (RT–qPCR)

Total RNA was first treated with genomic DNA (gDNA) at 36°C for 30 min to eliminate any genomic DNA. cDNA was synthesised using a cDNA synthesis kit (Takara Bio Inc., Kusatsu, Japan), according to the manufacturer's protocol. Quantitative PCR (qPCR) was conducted using SYBR Green Mix (Roche Diagnostics, Basel, Switzerland) and a QuantStudio 3 Real‐Time PCR System (Thermo Fisher Scientific Inc.), following the manufacturer's instructions. The qPCRs were carried out in a 20 μL system under the following conditions: an initial step at 95°C for 30 s, followed by 45 cycles of 95°C for 10 s, 60°C for 30 s and 72°C for 10 s. The relative expression levels of the target genes were calculated using the 2^−ΔΔCt^ method, with ACTB (beta‐actin) as the internal control. Primer information is provided in Table S2.

### 2.3 Western Blot

Cells were lysed using radioimmunoprecipitation assay (RIPA) buffer (cat. no. R0010; Solarbio), and the protein concentrations were measured using a BCA protein assay kit (Beijing Leagene Biotech Co. Ltd.). Protein samples (25 μg per lane) were separated by SDS–PAGE on 10% gels and transferred to PVDF membranes.

The membranes were blocked with protein‐free rapid blocking buffer (Epizyme Pharmaceutical Biotechnology Co. Ltd.) for 15 min at room temperature and incubated overnight at 4°C with primary antibodies against RBM6 (1:20,000, 14360‐1‐AP, proteintech), E‐cadherin (1:20,000, 20874‐1‐AP, proteintech), MMP16 (3 μg/mL, ab313410, abcam) and GAPDH (1:500, sc‐47724, Santa Cruz). After primary antibody incubation, membranes were incubated with either DyLight 800‐goat anti‐rabbit IgG (1:100, A23910, Abbkine) or DyLight 800‐goat anti‐mouse IgG secondary antibodies (1:100, A23920, Abbkine) for 1 h at 25°C. The membranes were then scanned using an imaging system (ODYSSEY CLx, Gene Company Limited) and the optical density was analysed using Image Studio Lite (LI‐COR Biosciences).

### 2.4 Transwell Invasion Experiment

The Transwell invasion chamber was prepared by diluting 50 mg/L Matrigel glue with medium in a 1:8 ratio, dripping it onto the upper part of the bottom membrane and drying at 4°C. After removing excess liquid, each well was hydrated with 50 μL of serum‐free medium at 37°C for 30 min. Target cells were starved in serum‐free medium for 1 day, digested with pancreatic enzyme, counted and adjusted to 3.5 × 10^5^/mL or 1.5 × 10^5^/mL for invasion or migration experiments, respectively. The adjusted cell suspension (200 μL/well) was added to the upper chamber. The lower chamber of the 24‐well plate contained 500 μL of medium with 20% serum. After incubating for 48 h, the upper layer of matrix glue and cells were gently removed, and the chamber was fixed in 5% paraformaldehyde, washed with PBS, stained with crystal violet and cleaned. The chamber was then placed on a slide for inverted microscope observation and imaging. ImageJ was used to count and analyse the collected images. Steps for the migration experiment were similar, differing only in the cell density adjustment.

### 2.5 RIP (RNA Binding Protein Immunoprecipitation)

The cells were collected, the nuclei were separated and cleaved, the fragmented chromatin was cleaved, the RBP antibody and the bound RNA were immunoprecipitated, and the bound RNA on the RBP after immunoprecipitation was washed and purified. The RNA was reversely transcribed into cDNA and analysed by qPCR.

### 2.6 RNA Pulldown

Design target RNA with biotin markers and then add magnetic beads to enrich RNA, the biotin‐labeled RNA is then used to interact with the RNA‐binding protein of interest under controlled conditions, ensuring sufficient time for the complex to form. Finally, the RNA‐protein complex was eluted from the magnetic beads. The elution sample is then prepared for Western blot analysis.

### 2.7 FISH (Fluorenscence in situ hybridization)

The probe was denatured in a 75°C constant temperature water bath. The specimens were roasted in an incubator at 50°C. The probe was added and hybridised. Wash away non‐specific binding probes. Signal amplification is performed when using biotin‐labelled probes. Undergo a nuclear staining. Make a seal. The results of FISH were observed by fluorescence microscope.

### 2.8 In Vivo Tumour Xenograft Model

All BALB/c‐nu mice were obtained from Sipeifu Company (Beijing, China). Approximately 1 × 10^5^ cells were were injected into the mice from the tail vein. After injection, the pinhole was gently pressed with the left index finger for about 1 min, and the mice were then returned to their cages. After the specified time, the mice were euthanized and the lung tissue was removed for further processing. All animal procedures were approved by the Ethics Committee of the First Affiliated Hospital of Zhengzhou University (2024‐KY‐1795).

### 2.9 Statistical Analysis

Statistical analyses were conducted via SPSS 22.0 (IBM, USA) and GraphPad Prism 9.3 (GraphPad Software Inc., USA). Qualitative data were presented as percentages. Quantitative data are expressed as mean ± standard deviation (SD) for normally distributed data or as interquartile range for non‐normally distributed data. Group comparisons were performed using *t*‐tests, one‐way analysis of variance or χ^2^ tests as appropriate. Pearson's correlation analysis was used to assess the relationships between the variables. All statistical tests were two‐tailed, and a *p*‐value of < 0.05 was considered to indicate statistical significance. All experiments were conducted in triplicate or more.

### 2.10 Ethics Approval and Consent to Participate

All animal procedures and use of human specimens were approved by the Ethics Committee of the First Affiliated Hospital of Zhengzhou University (2024‐KY‐1795).

## 3 Results

### 3.1 RBM6 Is Abnormally Expressed in Prostate Tumours and Negatively Correlated With Prognosis

**FIGURE 1:**
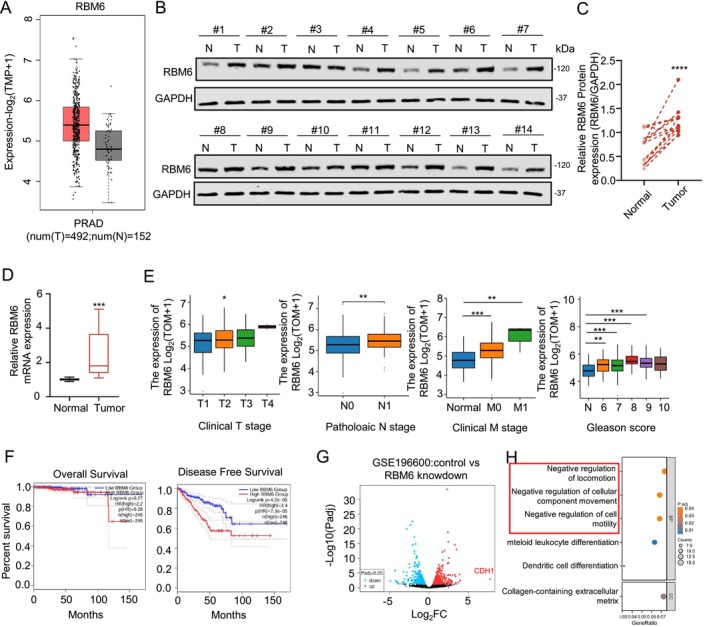
RBM6 is abnormally expressed in prostate tumours and negatively correlated with prognosis. (A) Expression of RBM6 in prostate tumours in TCGA database; (B) Expression levels of RBM6 protein in prostate tumours and normal specimens; (C) Statistical map of protein expression of RBM6 in prostate tumours and normal specimens; (D) RNA expression of RBM6 in prostate tumours and normal specimens; (E) Association between RBM6 and TNM staging and Glesason scores; (F) Relationship between RBM6 expression and prognosis of patients with prostate cancer; (G) Volcanic map of genetic changes after RBM6 knockdown; (H) Enrichment analysis of differential genes after RBM6 knockdown. n.s., no significance; *p < 0.05, **p < 0.01, ***p < 0.001, ****p < 0.0001.

We conducted an analysis of RBM6 expression in prostate tumours utilising the GEPIA database (http://gepia.cancer‐pku.cn/). Our findings revealed that RBM6 expression is elevated in tumours, indicating at its potential role as a cancer‐promoting factor in prostate cancer (Figure 1A). To further validate this observation, we collected tumour and adjacent non‐tumour specimens from 14 prostate cancer patients at the First Affiliated Hospital of Zhengzhou University and conducted PCR and WB experiments. These experiments confirmed that the expression level of RBM6 in prostate tumours is significantly higher compared to adjacent non‐tumour tissues (Figure 1B–D).

To gain a deeper understanding of the relationship between RBM6 and prostate cancer, we analysed the TCGA database. Our results indicated that RBM6 expression increases to varying degrees with advancing TNM stage, and patients with high RBM6 expression exhibit a poorer prognosis (Figure 1E,F). To further investigate how RBM6 impacts the prognosis of prostate cancer patients, we analysed the GEO database (GSE196600) and discovered a close correlation between RBM6 and changes in cell migration ability (Figure 1G,H).

### 3.2 RBM6 Promotes Cell Migration in Prostate Tumours

**FIGURE 2:**
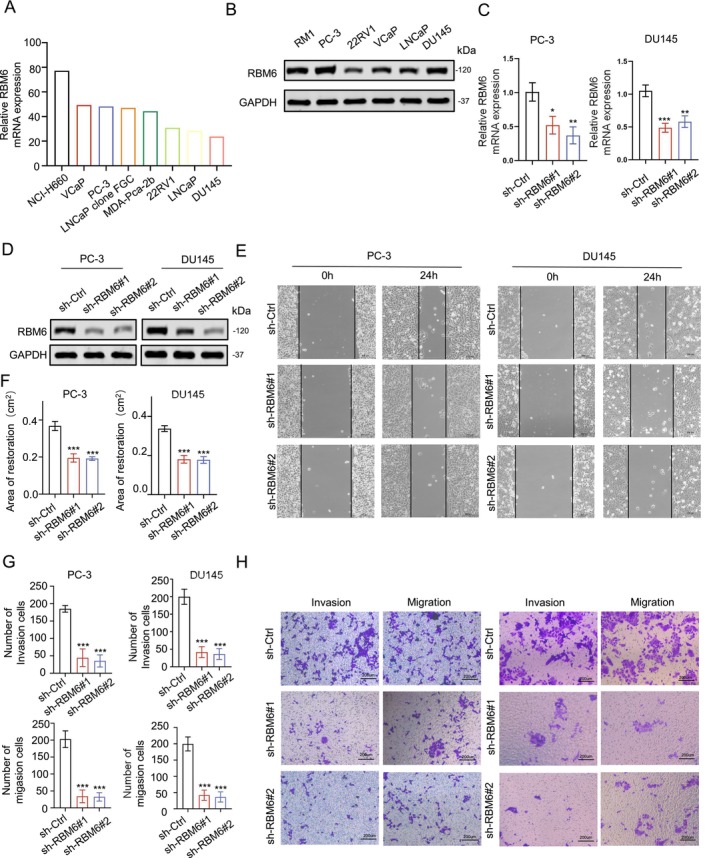
RBM6 promotes cell migration in prostate tumours. (A) RNA expression levels of RBM6 in different cell lines; (B) RBM6 protein expression levels in different cell lines; (C) RNA expression levels of RBM6 after the knockdown of RBM6; (D) Protein expression level of RBM6 after the knockdown of RBM6; (E, F) Wound healing experiment and healing area statistics after the knockdown of RBM6; (G, H) Transwell experiment and statistical analysis after the knockdown of RBM6. n.s., no significance; *p < 0.05, **p < 0.01, ***p < 0.001.

Based on the comprehensive biogenic analysis conducted earlier, we have deduced that RBM6 may exhibit a strong correlation with the cell migration capacity in prostate tissue. To further validate this hypothesis, we embarked on a series of functional experiments. By utilising the HPA database, we analysed the expression levels of RBM6 in prostate tumours and corroborated these findings through WB analysis. Our results demonstrated that RBM6 was highly expressed in the RM1, PC‐3 and DU145 prostate cancer cell lines (Figure 2A,B). Given their rapid growth characteristics, we selected the PC‐3 and DU145 prostate tumour cell lines for our subsequent experiments.

Initially, we conducted PCR and WB experiments to assess the impact of the shRNA knockdown plasmid on RBM6 expression (Figure 2C,D). Our scratch assays revealed that knocking down RBM6 significantly reduced the migratory ability of the cells after 24 h (Figure 2E,F). Similarly, transwell migration and invasion tests further confirmed that the number of cells capable of traversing the transwell chamber was markedly decreased when RBM6 was knocked down (Figure 2G,H). Collectively, these findings strongly suggest that the knockdown of RBM6 can effectively inhibit the migration of prostate tumour cells.

### 3.3 RBM6 Promotes Cell Metastasis Primarily Through Inhibition of CDH1

**FIGURE 3:**
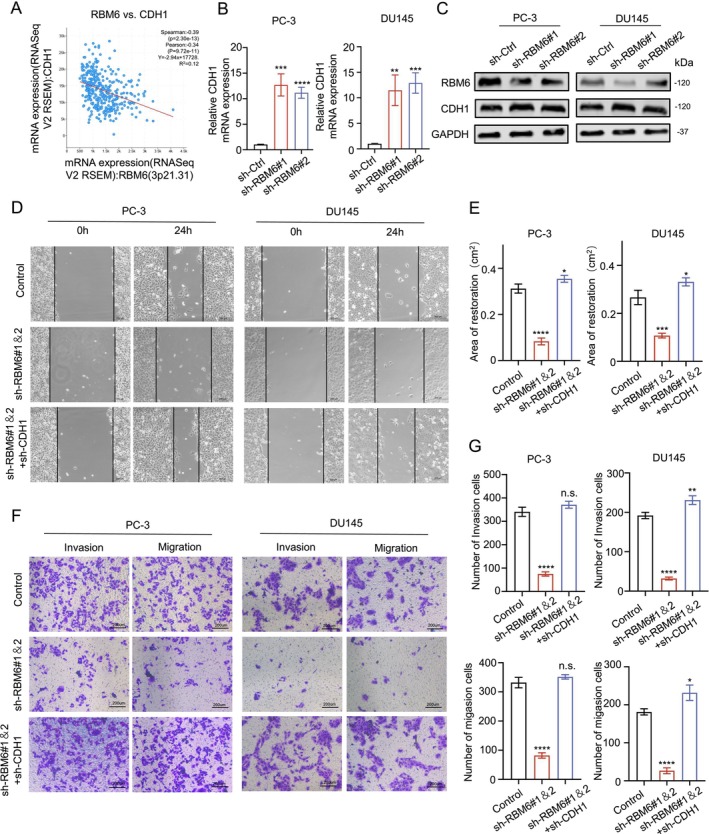
RBM6 promotes cell metastasis primarily through inhibition of CDH1. (A) Correlation graph between RBM6 and CDH1; (B) RNA expression levels of CDH1 after the knockdown of RBM6; (C) Protein expression levels of CDH1 after the knockdown of RBM6; (D, E) Wound healing experiment and healing area statistics after the knockdown of either RBM6 alone or both RBM6 and CDH1 together; (F, G) Transwell experiment and statistical analysis after the knockdown of either RBM6 alone or both RBM6 and CDH1 together; n.s., no significance; *p < 0.05, **p < 0.01, ***p < 0.001, ****p < 0.0001.

Through the analysis of GEO data, we found that CDH1 (one of the most obvious characteristics of cell migration ability changes [15]) was significantly increased after RBM6 knockdown (Figure 1G). Therefore, we hypothesized that the promotion effect of RBM6 on the migration ability of prostate tumours may be played by CDH1. Therefore, we first analysed the relationship between RBM6y and CDH1 in the database, and the results showed that RBM6 and CDH1 were significantly negatively correlated (Figure 3A). Then, we also verified by PCR and WB that the expression level of CDH1 was changed after RBM6 overexpression, and the results suggested that the expression of CDH1 was significantly increased after RBM6 knockdown (Figure 3B,C). In order to further verify the role of CDH1 in the function of RBM6, we set the control group, the RBM6 knockdown group and the RBM6 knockdown group at the same time. The group of RBM6 knockdown found that RBM6 knockdown could inhibit the migration ability of cells, while the simultaneous knockdown of CDH1 could inhibit this effect of RBM6 (Figure 3D–G), related gene expression is shown in the supplementary map (Figure S3C,D). According to the reported literature [16], RBM6 is likely to be regulated by binding with CDH1 RNA. Therefore, we designed the probe of CDH1 to conduct RNA pull down experiment and RIP experiment, and found that CDH1 and RBM6 have binding relationship (Figure S1A–C).

### 3.4 RBM6 Is Regulated by ZEB1 and Inhibits ZEB1‐Induced Cell Migration

**FIGURE 4:**
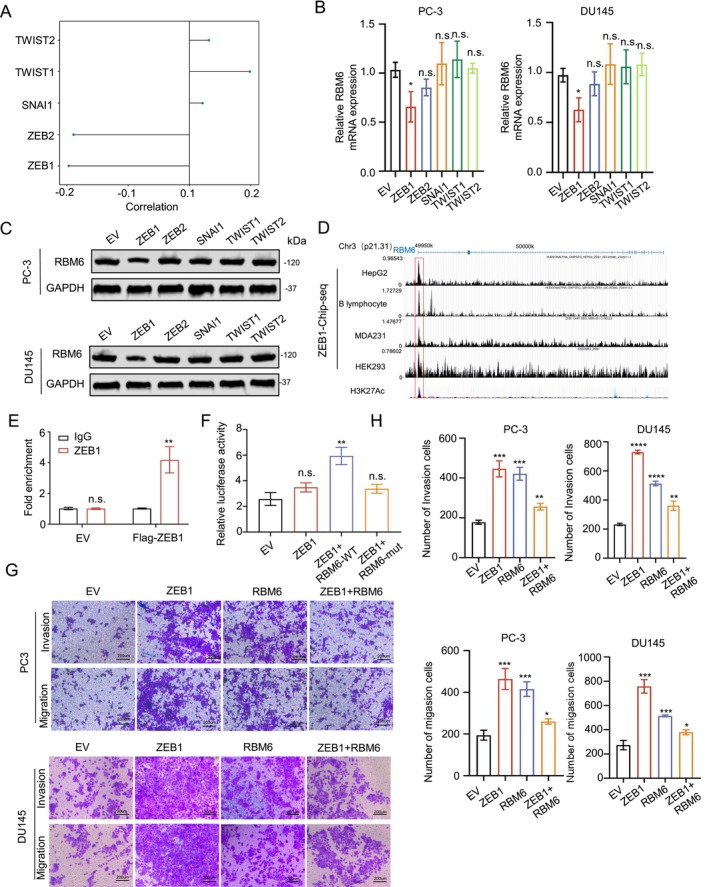
RBM6 is regulated by ZEB1 and inhibits ZEB1‐induced cell migration. (A) Lollipop chart of correlation between EMT pathway transcription factors and RBM6; (B) RNA expression levels of RBM6 after overexpression of related genes; (C) Protein expression levels of RBM6 after overexpression of related genes; (D) ZEB1 chipseq results at the RBM6 promoter position; (E) Chip‐qPCR experiment; (F) Double luciferase experiment; (G, H) Transwell experiment and statistical analysis after the overexpression of ZEB1, RBM6 or a combination of both. n.s., no significance; *p < 0.05, **p < 0.01, ***p < 0.001, ****p < 0.0001.

The EMT pathway, which is closely related to the ability of tumour metastasis, has been reported to play an important role in the occurrence and development of prostate tumours [17]. Having clarified the relationship between RBM6 and CDH1, we next explored whether RBM6's ability to regulate cell migration is associated with the EMT pathway. To investigate this, we analysed the correlation between key transcription factors in the EMT pathway—ZEB1, ZEB2, TWIST and SNAI1—and RBM6 using prostate cancer data from the cBioPortal database (Prostate Adenocarcinoma, TCGA, Cell 2015, https://www.cbioportal.org/). Our analysis revealed potential regulatory relationships between RBM6 and both ZEB1 and ZEB2 (Figure 4A).

To further investigate these interactions, we examined the expression changes of RBM6 upon overexpressing vectors of these key transcription factors. Our findings indicated that the expression of RBM6 was specifically inhibited when ZEB1 was highly expressed (Figure 4B,C). To validate the regulatory relationship between ZEB1 and RBM6, we searched ChIP‐SEQ data for ZEB1 on the Cistrome website and discovered that ZEB1 exhibited varying degrees of binding peaks in the promoters and adjacent regions of RBM6 across multiple datasets (Figure 4D). We subsequently confirmed the binding between ZEB1 and RBM6 through ChIP‐qPCR and dual luciferase reporter experiments (Figure 4E,F).

Given that both ZEB1 and RBM6 are known to promote cell migration, but our experiments revealed a negative transcriptional regulation between them, we conducted transwell experiments to assess the impact of simultaneous overexpression of RBM6 and ZEB1 on cell migration. Our results indicated that, in the presence of high ZEB1 expression, co‐transfection with the RBM6 overexpression plasmid inhibited ZEB1‐induced cell migration (Figure 4G,H), related gene expression is shown in the supplementary map (Figure S3E,F). This intriguing finding suggests that RBM6 may exhibit a functional reversal when ZEB1 is highly expressed, transitioning from a promoter of migration to an inhibitor of migration.

### 3.5 The Reversal of RBM6's Function Is Linked to the Regulation of Substrate Proteins

**FIGURE 5:**
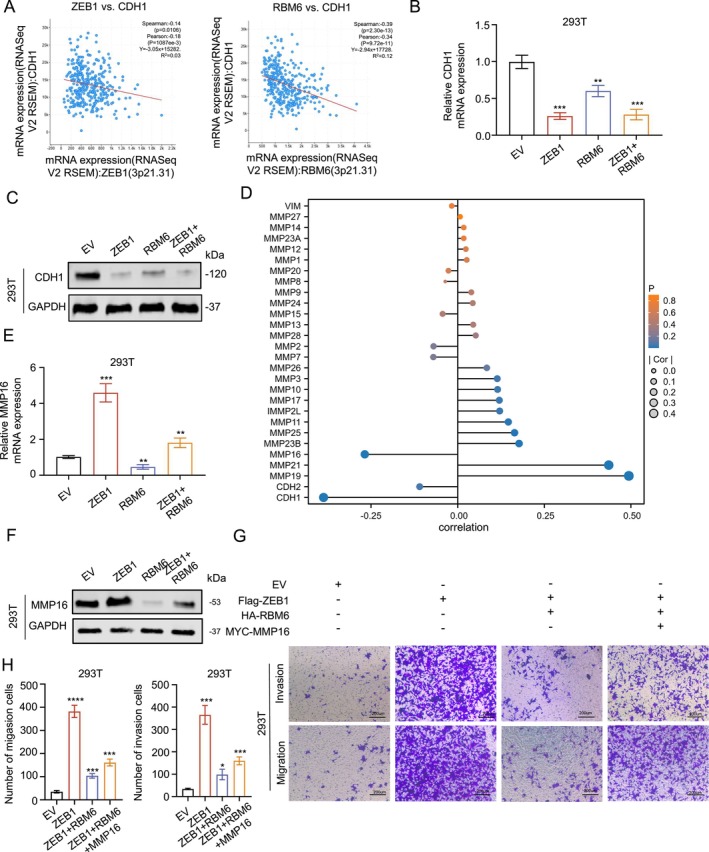
The reversal of RBM6's function is linked to the regulation of substrate proteins. (A) Correlation diagram of CDH1 with ZEB1 and RBM6; (B) RNA expression levels of CDH1 after the overexpression of ZEB1, RBM6 or a combination of both; (C) Protein expression levels of CDH1 after the overexpression of ZEB1, RBM6 or a combination of both; (D) Lollipop chart of correlation between important substrates of EMT pathway and RBM6; (E) RNA expression levels of MMP16 after the overexpression of ZEB1, RBM6 or a combination of both; (F) Protein expression levels of MMP16 after the overexpression of ZEB1, RBM6 or a combination of both; (G, H) Transwell experiment and statistical analysis after simultaneous overexpression of ZEB1 alone, ZEB1 with RBM6, and ZEB1 with both RBM6 and MMP16. n.s., no significance; *p < 0.05, **p < 0.01, ***p < 0.001, ****p < 0.0001.

Next, we set out to explore why RBM6 is reversed in the presence of high ZEB1 expression. According to relevant literature reports, ZEB1 can promote the occurrence of EMT by inhibiting the expression of CDH1 [18]. Through the analysis of the correlation between RBM6 and EMT‐effector proteins, we found that RBM6 is also significantly negatively correlated with CDH1, which may be an important mechanism of RBM6 to promote migration (Figure 5A). Then we further verified the conjecture by PCR and WB, and found that both ZEB1 and RBM6 could inhibit CDH1, but when ZEB1 was highly expressed, the inhibitory effect of RBM6 on CDH1 was not obvious (Figure 5B,C). Interestingly, when we analysed the relationship between RBM6‐ and EMT‐effector proteins, we found that it was closely related to MMP family proteins, while MMP16, as a protein that promotes migration [19], was negatively related to RBM6 (Figure 5D).

So we hypothesized: When ZEB1 is highly expressed, the migration‐related effector protein similar to CDH1 is significantly inhibited, resulting in the increased expression of RBM6, which cannot promote migration through further inhibition of CDH1, while the expression of MMP16 is significantly increased due to the high expression of ZEB1. At this time, the inhibitory effect of RBM6 on MMP16 is more prominent under this condition, thus exerting the function of inhibiting migration.

In order to verify this hypothesis, we found through PCR and WB experiments that overexpression of ZEB1 can significantly enhance MMP16, while overexpression of RBM6 can inhibit MMP16, and the inhibition effect is more obvious in the case of overexpression of ZEB1(Figure 5E,F), these findings were also replicated in PC‐3 and DU145 cell lines (Figure S2A,B). Similarly, in transwell migration and invasion experiments, we found that RBM6 was able to resist ZEB1‐induced cell migration, but the inhibitory effect of RBM6 was significantly reduced when MMP16 was overexpression (Figure 5G,H), the same phenomenon was also demonstrated in PC‐3 and DU145 cell lines (Figure S2C). Both MMP16 and CDH1 play an important role in the function of RBM6 to influence cell migration (Figure S3A,B). Through the above experiments, we found that the reversal of RBM6 function may be caused by the expression of MMP16 under different conditions.

### 3.6 The RBM6 Protein Binds to MMP16 in the Nucleus

**FIGURE 6:**
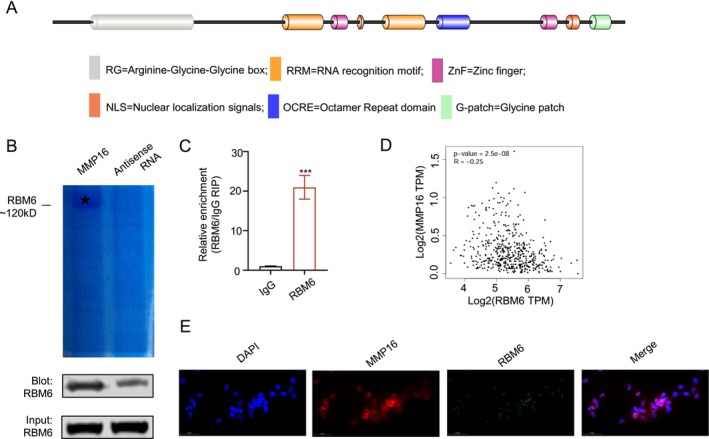
The RBM6 protein binds to MMP16 in the nucleus. (A) RBM6 gene structure diagram; (B) Coomassie bright blue staining and WB after RNA pulldown; (C) RIP experiment; (D) Correlation between RBM6 and MMP16; (E) FISH co‐localization experiment of RBM6 and MMP16. ***p < 0.001.

Based on the RBM6 structure described in the relevant literature, we identified its domain, with the RNA‐binding domain being a crucial component for RNA binding (Figure 6A). To further investigate the regulatory mechanism between RBM6 and MMP16, we developed an MMP16 probe for an RNA pull‐down experiment, which successfully pulled down the MMP16 protein. Additionally, we observed a significant increase in MMP16 enrichment during the RIP experiment (Figure 6B,C). Concurrently, we corroborated their relationship using data from the TCGA database (Figure 6D). The results of the FISH co‐localization experiment further confirmed the co‐localization of RBM6 and MMP16 in the nucleus (Figure 6E).

### 3.7 RBM6 Can Inhibit Cell Migration in LNCaP Cells With Drug Resistance

**FIGURE 7:**
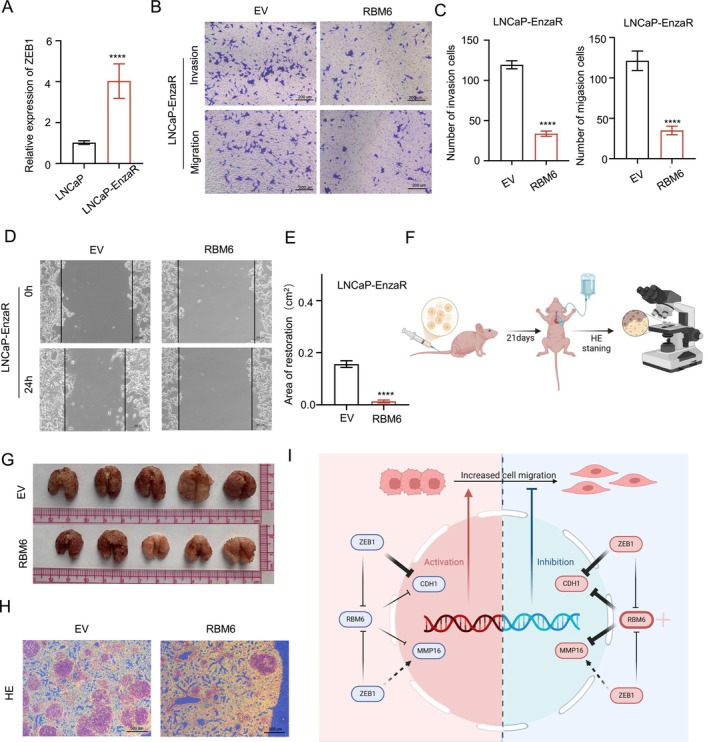
RBM6 can inhibit cell migration in LNCaP cells with drug resistance. (A) RNA expression levels of ZEB1; (B, C) Transwell experiment and statistical analysis after overexpression of RBM6; (D, E) Wound healing experiment and healing area statistics after overexpression of RBM6; (F) Mouse lung perfusion diagram; (G) General picture of mouse lung organs; (H) HE staining of the largest transverse section of the lung; (I) Mechanism diagram. ****p < 0.0001.

Recent studies have reported that there may be high expression of ZEB1 in enzalutamide resistant lines [20]. So we also verified it in the enzalutamide resistant lines we constructed (Figure 7A). Simultaneously, we conducted an analysis of the expression levels of RBM6, CDH1, MMP16 and other genes in drug‐resistant cell lines. The results revealed a significant downregulation of RBM6 and a significant upregulation of MMP16 in these drug‐resistant lines, whereas the expression of CDH1 remained relatively unchanged (Figure S1D). We overexpressed RBM6 in the LNCaP cell line, and transwell experiment showed that the migration and invasion of RBM6 cells decreased significantly after overexpression (Figure 7B,C). Cell migration experiments showed the same results (Figure 7D,E). We simultaneously examined the expression levels of CDH1 and MMP16 at this point, suggesting that the migration‐inhibiting effect of RBM6 in drug‐resistant cell lines is caused by abnormal expression of MMP16 (Figure S2D,E). To further demonstrate the role of RBM6 in drug‐resistant lines, we constructed a lung metastasis model using tail vein injection in nude mice to determine the ability of cells to migrate in vivo (Figure 7F). The results also suggest that the number of lung metastases in mice can be significantly reduced after RBM6 overexpression (Figure 7G,H).

In summary, we have uncovered that RBM6 exhibits the capability to enhance cell migration through the inhibition of CDH1. However, in the presence of elevated ZEB1 expression, the functional role of RBM6 is reversed. This reversal arises because, under such conditions, ZEB1's inhibitory action on CDH1 and its stimulatory effect on MMP16 diminish RBM6's inhibitory influence on CDH1 while accentuating its inhibitory effect on MMP16, thereby demonstrating its potential to suppress migration. It is noteworthy that ZEB1 has been documented to be overexpressed in drug‐resistant cell lines, implying that RBM6 may represent a promising therapeutic target for patients with drug resistance and castration‐resistant prostate cancer (CRPC) (Figure 7I).

## 4 Discussion

RBM6 has previously been reported to play an important role in a variety of tumours. For example, Wang et al. [13] reported that RMB6 could inhibit laryngeal cancer, while Bechara et al. [14] reported that RBM6 could promote the proliferation of lung cancer, which reflected the uncertainty of RBM6 in cancer research. In this study, we observed that RBM6 is abundantly expressed in prostate tumours (Figure 1A–D) and exhibits a negative correlation with patient prognosis (Figure 1F). Furthermore, our findings indicate that RBM6 has the capacity to enhance the migratory abilities of prostate tumour cells (Figure 2). This indicates the promising potential of RBM6 as both a predictive biomarker and a therapeutic target in prostate tumours.

In this study, we observed an intriguing phenomenon that warrants further exploration: the multi‐faceted functionality of the molecule RBM6, which exhibits distinctly different effects under various conditions. Previous research has established that RBM6 can enhance cell migration in specific contexts, offering fresh insights into the mechanisms underlying cell motility. Our findings reveal that RBM6 promotes the migratory capacity of prostate tumours by inhibiting CDH1, and its activity is also regulated by the key transcription factor ZEB1 of the EMT pathway (Figures 3 and 4). This discovery not only deepens our understanding of the EMT regulatory network but also underscores its complexity. It further identifies a potential target for developing novel prostate cancer treatment strategies.

However, as our study progressed, a significant functional reversal occurred: when ZEB1 expression levels were elevated, the function of RBM6 changed dramatically from promoting cell migration to inhibiting cell migration. Our results confirm that since ZEB1 itself possesses the ability to effectively inhibit CDH1, in this scenario, the inhibitory effect of RBM6 on CDH1 is diminished. Meanwhile, the elevated expression of ZEB1 results in a notable increase in the expression of MMP16. Consequently, in this context, the inhibitory effect of RBM6 on MMP16 becomes more pronounced, ultimately leading to a decrease in cell migration ability (Figure 5). In Figure 7, it was observed that RBM6 exhibits inhibitory effects on tumour migration in enzalutamide‐resistant LNCaP cell lines, indicating at the significant potential of RBM6 as a therapeutic target for patients suffering from advanced Castrate‐Resistant Prostate Cancer (CRPC). This finding highlights the importance of considering the conditional dependence of molecular function in areas such as drug development and gene therapy to ensure the effectiveness and safety of therapeutic approaches.

As a member of the RBP protein that can bind to RNA, RBM6 functions by participating in RNA metabolism, including splicing, transport, translation and stability [16, 21, 22]. It has been reported that RBM5 and RBM6 can bind to exon regions near splicing sites and recruit splicing components [14], RBM6 can also promote the mRNA stability [23]. In this study, we found that RBM6 can negatively regulate its stability by binding to the RNA of MMP16 (Figure 5F). However, the specific how RBM6 combines with MMP16 and through what pathway can inhibit its RNA stability is limited by some conditions, which could not be further explored in this experiment.

In conclusion, this study demonstrates that RBM6 displays diverse functional properties in different environments and provides insights into its precise molecular mechanism. It suggests that RBM6 could be a promising therapeutic target under specific conditions. The results also emphasise the importance of considering the condition specificity of molecular function in drug development and gene therapy to ensure effectiveness and safety.

## Author Contributions

**Ruoyang Liu:** conceptualization (equal), investigation (equal), writing – review and editing (equal). **Yu Liu:** data curation (equal). **Long Zhang:** investigation (equal), methodology (equal). **Xiang Li:** investigation (equal), methodology (equal), supervision (equal), validation (equal). **Ningyang Li:** data curation (equal), writing – original draft (equal). **Fubo Lu:** formal analysis (equal), resources (equal). **Wansheng Gao:** data curation (equal), formal analysis (equal), project administration (equal). **Zhankui Jia:** funding acquisition (equal). **Zhenlin Huang:** funding acquisition (equal). **Jinjian Yang:** investigation (equal), visualization (equal).

## Ethics Statement

All animal procedures were approved by the Ethics Committee of the First Affiliated Hospital of Zhengzhou University (2024‐KY‐1795).

## Conflicts of Interest

The authors declare no conflicts of interest.

## Supporting information


**Data S1.** Supporting Information.

## Data Availability

The data that supports the findings of this study are available in the Supporting Information of this article.
