# Supplementary material for: The Oncopromoting Gene RBM6 Inhibits Prostate Tumour Cell Migration During Epithelial‐to‐Mesenchymal Transition
Source: J Cell Mol Med. 2025 Feb 3;29(3):e70397. doi: 10.1111/jcmm.70397 (PMC11790351; doi:10.1111/jcmm.70397)
Supplement: Supplementary file 1 — Data S1. Supporting Information. [file JCMM-29-e70397-s001.docx]

**Supplement fig1: RBM6 can inhibit CDH1 expression at the transcriptional level**

**
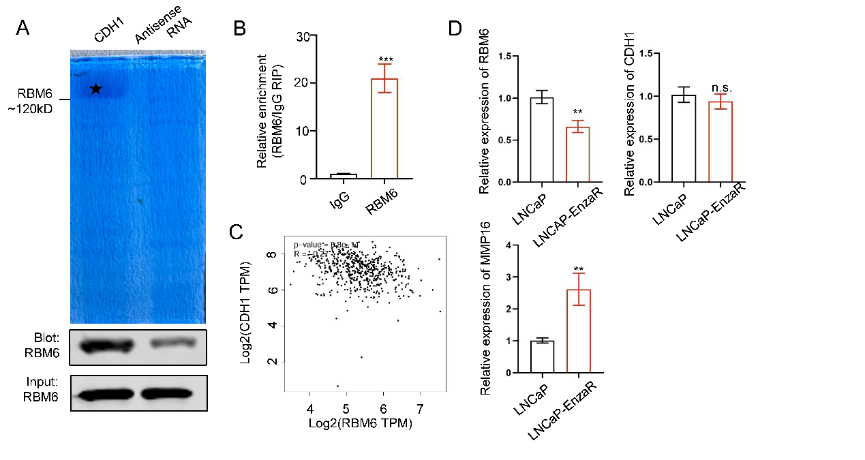
**

A: Coomassie bright blue staining and WB after RNA pulldown; B: RIP experiment; C: RBM6 and CDH1 correlation graph; D: RNA expression levels of RBM6, CDH1 and MMP16 in LNCaP-EnzaR cell line. n.s., no significance; ***P* < 0.01, ****P* < 0.001

**Supplement fig2: MMP16 plays a significant role in the reversal of RBM6 function
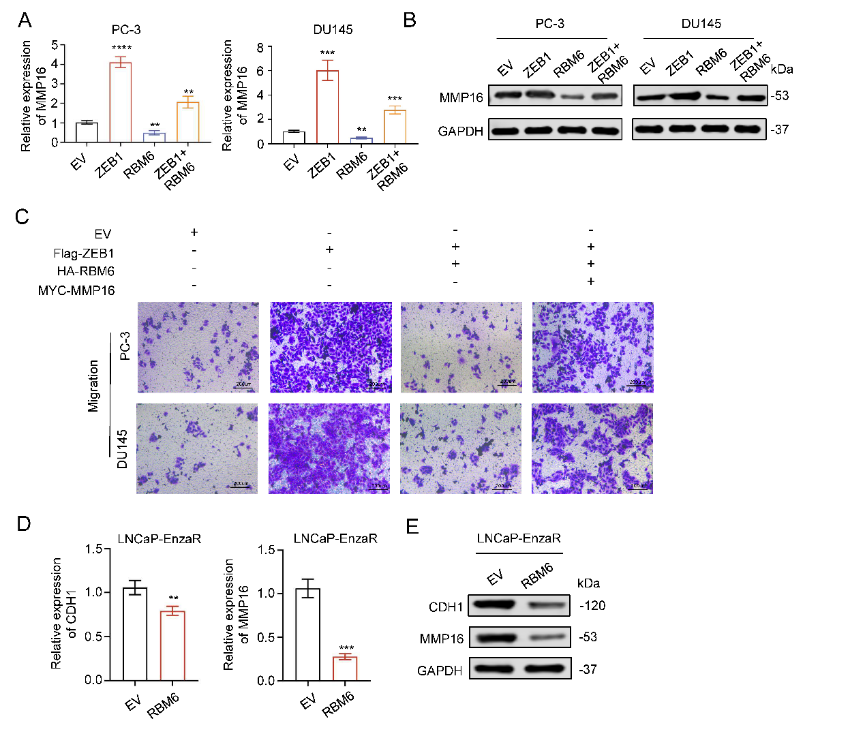
**

A: The expression level of MMP16 transcription level after ZEB1 and RBM6 overexpression treatment; B: The expression level of MMP16 protein after ZEB1 and RBM6 overexpression treatment; C: Transwell experiments in PC3 and DU145 after simultaneous overexpression of ZEB1 alone, ZEB1 with RBM6, and ZEB1 with both RBM6 and MMP16; D: The expression level of CDH1 and MMP16 transcription level after RBM6 overexpression treatment; E: The expression level of CDH1 and MMP16 protein after RBM6 overexpression treatment . ***P* < 0.01, ****P* < 0.001, *****P* < 0.0001

**Supplement fig3:** **CDH1 and MMP16 play an important role in the functioning of RBM6**


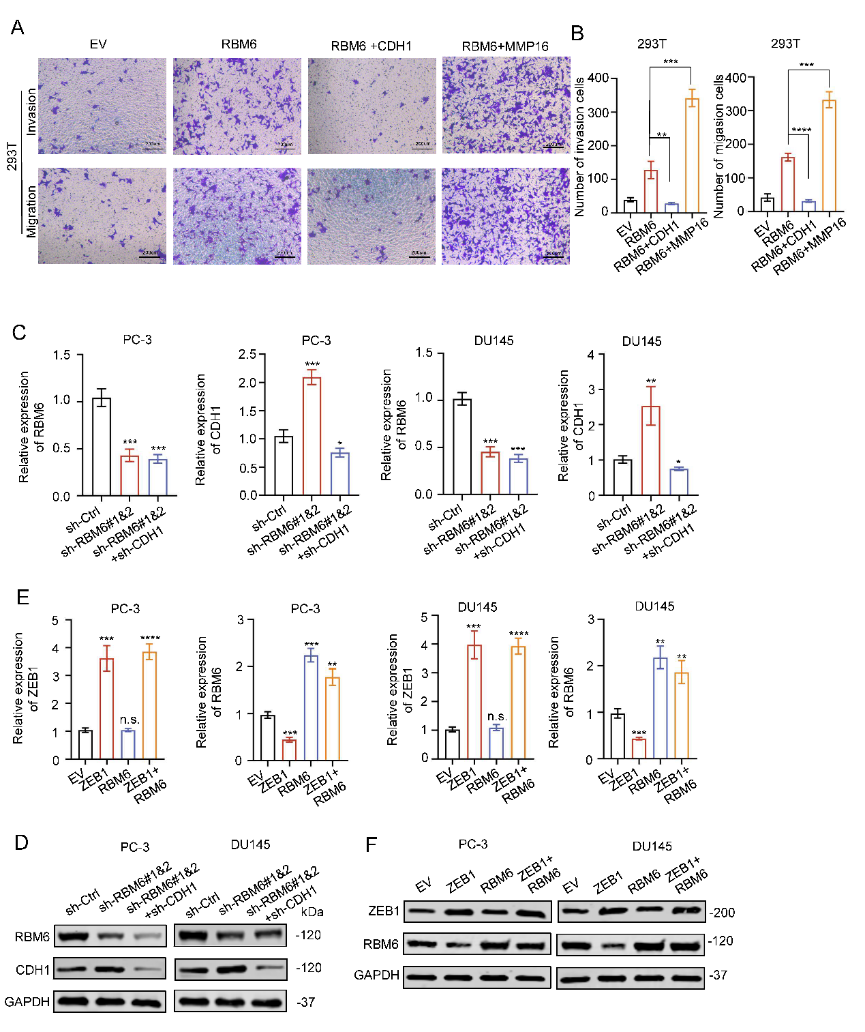


A and B: Transwell experiments in 293T after simultaneous overexpression of RBM6 alone, RBM6 with CDH1, and RBM6 with MMP16; C: Transcription expression levels of RBM6 and CDH1 after the knockdown of either RBM6 alone or both RBM6 and CDH1 together; D: Protein expression levels of RBM6 and CDH1 after the knockdown of either RBM6 alone or both RBM6 and CDH1 together; E: Transcription expression levels of RBM6 and ZEB1 after the overexpression of ZEB1, RBM6, or a combination of both; F: Protein expression levels of RBM6 and ZEB1 after the overexpression of ZEB1, RBM6, or a combination of both; **P* < 0.05,***P* < 0.01, ****P* < 0.001, *****P* < 0.0001

**Additional Components for Submission**

**Graphical Abstract**

**
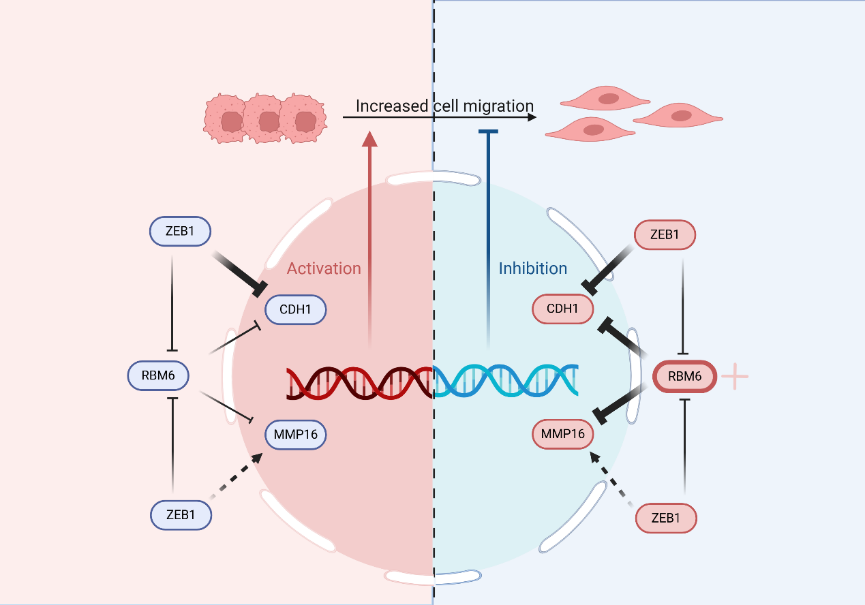
**

RBM6 exhibits the capability to enhance cell migration through the inhibition of CDH1. However, in the presence of elevated ZEB1 expression, the functional role of RBM6 is reversed. This reversal arises because, under such conditions, ZEB1's inhibitory action on CDH1 and its stimulatory effect on MMP16 diminish RBM6's inhibitory influence on CDH1 while accentuating its inhibitory effect on MMP16, thereby demonstrating its potential to suppress migration.

**Supplementary Table 1.** **The oligonucleotides of siRNA or shRNA.**

| Product name | Forward primers (5’-3’) |
| --- | --- |
| RBM6 shRNA-1 | GAAGGAGTATAACACAGGTTA |
| RBM6 shRNA-2 | CAAAGAAGTTACCCTGGAGTA |
| CDH1 shRNA-1  CDH1 shRNA-2 | CCAAGCAGAATTGCTCACATT  CGATTCAAAGTGGGCACAGAT |

* The oligonucleotides from WeizhenBio, Shandong, China.

**Supplementary Table 2. Oligonucleotides used for relative gene expression by qRT-PCR.**

| Target gene | Forward primers (5’→3’) | Reverse primers (5’ →3’) |
| --- | --- | --- |
| RBM6 | TGGAGTATGTATCAAGCCTGGA | ATGAACAGGAAGATCGGTGCC |
| CDH1 | CGAGAGCTACACGTTCACGG | GGGTGTCGAGGGAAAAATAGG |
| MMP16 | AGCACTGGAAGACGGTTGG | CTCCGTTCCGCAGACTGTA |
| ZEB1 | GATGATGAATGCGAGTCAGATGC | ACAGCAGTGTCTTGTTGTTGT |
| ACTB | CCTGGCACCCAGCACAAT | GGGCCGGACTCGTCATAC |
